# Capturing sex differences in spontaneous autonomic fluctuations of resting heart rate using a similarity graph theory approach

**DOI:** 10.1186/s13293-026-00904-x

**Published:** 2026-04-25

**Authors:** Lin Sørensen, Elisabet Kvadsheim, Julian Koenig, Julian F. Thayer, DeWayne P. Williams, Hayley J. MacDonald, Ryan Douglas McCardle, Daniel Wollschlaeger, Ole Bernt Fasmer, Berge Osnes

**Affiliations:** 1 Department of Clinical and Biological Psychology, University of Bergen, Bergen, Norway; 2 Division of Mental Health and Addiction, Oslo University Hospital, Oslo, Norway; 3 Department of Child and Adolescent Psychiatry, Psychosomatics, and Psychotherapy, Faculty of Medicine and University Hospital Cologne, University of Cologne, Cologne, Germany; 4 Department of Psychological Science, University of California, Irvine, CA USA; 5 Department of Psychology, Ohio State University, Columbus, OH USA; 6 Institute of Medical Biostatistics, Epidemiology and Informatics, University Medical Centre, Johannes Gutenberg-University, Mainz, Germany; 7 Department of Clinical Medicine, University of Bergen, Bergen, Norway

**Keywords:** Heart rate variability, Graph theory, Short-term fluctuations, Inter-beat-intervals of the heart, Sex differences

## Abstract

**Background:**

Autonomic control of the heart is an important indicator of self-regulation and overall mental and physical health. The vagus nerve plays a central role in this regulation, and resting-state heart rate variability (HRV), reflecting fluctuations in inter-beat intervals (IBIs), is the primary noninvasive marker of vagal activity. As males and females differ in aspects of self-regulation, HRV may help elucidate underlying neurobiological differences. However, sex differences in commonly used HRV metrics, such as natural log transformed root mean square of successive RR interval differences (lnRMSSD) and high-frequency HRV (lnHF-HRV) derived from 5-minute recordings, appear smaller in young adults than in other age groups. These metrics capture vagally mediated activity as averaged linear measures and may therefore overlook rapid, spontaneous IBI fluctuations. In the present study, we tested whether a similarity graph theory algorithm could better capture sex differences in nonlinear, rapid IBI variability within 2–5-seconds time windows.

**Methods:**

Electrocardiogram (ECG) recordings of 269 young, healthy adults between 18 and 30 years old (M = 21.5, SD = 3.0) were pooled from three different studies. Males accounted for 52.4% of participants, indicating a comparable distribution between sexes. Similarity graph–theory metrics were computed to quantify nonlinear, rapid interbeat interval (IBI) variability using sliding windows of 2–5 s and ≥12 s. In addition, conventional linear and nonlinear heart rate variability metrics, including lnRMSSD and lnHF-HRV, were calculated. Logistic regression models were used to assess the predictive value of graph-theory and HRV metrics for sex, both separately and in combined models for comparison. All models were adjusted for age, body mass index, mean heart rate, and respiratory rate.

**Results:**

Males showed higher graph-metric values, indicating lower IBI variability compared with females (odds ratio 2.78; 95% CI 1.32–5.86). Neither lnRMSSD nor lnHF-HRV distinguished sexes alone; however, lnRMSSD became predictive when combined with the graph metric (odds ratio 1.73; 95% CI 1.06–2.81), although this effect was attenuated after controlling for mean heart rate.

**Conclusions:**

These findings suggest that nonlinear methods sensitive to rapid spontaneous IBI changes can complement traditional short-term HRV metrics for assessing sex differences in autonomic heart regulation.

**Supplementary Information:**

The online version contains supplementary material available at 10.1186/s13293-026-00904-x.

## Abstract (plain language summary)

The way our heart adjusts from beat to beat tells us a lot about how well we can adapt to stress, emotions, and everyday demands. This natural variation in heartbeats is called heart rate variability (HRV) and is usually measured using sensors that record the heart’s electrical activity. Men and women often differ in how they regulate emotions and behavior, and these differences can sometimes be seen in HRV. In many studies, women tend to show more flexible heart activity than men. However, in young adults, these differences are often hard to detect using standard methods. This may be because young hearts are especially adaptable and change quickly in response to the situation. To address this, we used a new method that looks at very short-term changes in heartbeat patterns using a mathematical approach called graph theory. This method was able to detect differences that traditional measures missed, showing that young women had more flexible heart activity than young men when these rapid fluctuations were taken into account.

**Supplementary Information:**

The online version contains supplementary material available at 10.1186/s13293-026-00904-x.

## Highlights

We examined sex differences in rapid, spontaneous heart rate fluctuations using a graph-theory–based algorithm.

This approach captured nonlinear interbeat-interval variability within short (2–5 s) time windows.

Graph-theory metrics were compared with conventional heart rate variability metrics.

Only the graph-theory metric detected sex differences in heart rate variability in young adults.

**Supplementary Information:**

The online version contains supplementary material available at 10.1186/s13293-026-00904-x.

## Introduction

Autonomic control of the heart is an important marker of both somatic and mental health, reflecting the capacity to adapt to stress and environmental demands [1, 2]. This regulation is commonly assessed using heart rate variability (HRV), which quantifies fluctuations in inter-beat intervals (IBIs) [1]. Higher HRV is associated with enhanced emotional and cognitive regulation [3–5], whereas lower HRV is observed in individuals with mental health disorders [6, 7], and predicts an increased risk of cardiovascular disease and all-cause mortality [8, 9].

Central to these processes is the vagus nerve, which plays a key role in autonomic regulation, with HRV serving as a primary noninvasive index of vagus nerve activity [1, 10]. Research employing HRV as a psychophysiological marker of self-regulatory traits has increasingly focused on potential sex differences in resting-state HRV [11, 12]. This focus is supported by evidence suggesting sex differences in emotional and behavioral self-regulation, although these vary depending on the domain assessed and the stage of life [13]. In this context, examining sex differences in the autonomic regulation of heart rate may contribute to a broader understanding of self-regulatory processes. However, it is important to note that these associations are indirect and context-dependent and do not imply a one-to-one relationship with behavioral or clinical outcomes. Consistent with this framework, meta-analytic findings indicate that adult females generally exhibit higher HRV than males [12]. Although these sex differences in HRV become more pronounced with increasing age, such a pattern is not observed in younger adult populations. Given the central role of vagal function in autonomic regulation, further investigation of sex differences in vagally mediated IBIs may improve our understanding of physiological regulation and support more personalized approaches in healthcare [14, 15].

Cardiac autonomic regulation reflects the dynamic balance between the parasympathetic and sympathetic branches of the autonomic nervous system [1]. The vagus nerve, via the parasympathetic pathway, enables rapid adjustments in heart rate and IBIs [16]. Parasympathetic effects occur within milliseconds, whereas sympathetic influences peak after about five seconds [17]. Vagal activity acts almost immediately, producing changes in IBIs within one to two heartbeats [18]. vmHRV is commonly indexed by resting-state metrics of high-frequency HRV (HF-HRV; 0.15–0.40 Hz) and the time-domain metric root mean square of successive RR interval differences (RMSSD) [10, 16, 18], both reflecting short-term variability in IBIs and parasympathetic modulation of heart rate. Higher values indicate greater vagal activity. Although these metrics are highly correlated [19, 20], RMSSD appears less influenced by respiration rate [21]. Meta-analytic evidence across age groups shows sex differences in raw resting HF-HRV but only a trend for resting RMSSD [12], with no significant differences after natural log transformation (e.g., lnRMSSD and lnHF-HRV) [12, 22] .

Rapid, spontaneous fluctuations in heart rate may not be fully captured by traditional resting vmHRV metrics such as lnHF-HRV and lnRMSSD. These measures rely on linear models and averaged statistics from relatively long ECG recordings (typically 1–5 min), which may obscure subtle differences in IBI organization between females and males. As a result, vmHRV values can appear similar across sexes despite underlying differences in vagal dynamics. This limitation may be particularly relevant in adults under 40, who show greater IBI complexity and nonlinear organization than older adults [23]. Consistent with this, meta-analytic findings indicate that sex differences in RMSSD increase with age [12], possibly reflecting a shift toward more linear vagal regulation over time, with evidence suggesting this shift begins after age 30 [24]. Accordingly, studies of young adults (18–30 years) generally report no sex differences in lnHF-HRV or lnRMSSD [25–28].

Biological evidence suggests sex-specific vagal organization of IBIs, influenced both directly by sex hormones (e.g., estrogen, testosterone) [29, 30] and indirectly through their modulation of monoamine neurotransmitters [31]. For example, estradiol increases striatal dopamine release [32], which in turn modulates autonomic cardiac control and contributes to both tonic and rapid heart rate fluctuations [33, 34]. Although females may exhibit higher vmHRV, rapid IBI variations are likely better captured by nonlinear analytical methods. Such methods have been used to assess IBI organization and sex differences in HRV [35–37], with mixed findings [12]. Metrics of complexity and regularity, such as sample entropy, approximate entropy, and detrended fluctuation analysis (DFAα1), have been shown to distinguish females from males, whereas the short-term HRV metric SD1 has not. However, these measures are typically derived from longer ECG recordings, which may limit their sensitivity to rapid dynamics, and can be difficult to interpret without assumptions about underlying system dynamics [38]. This highlights the need for nonlinear approaches that more specifically capture rapid and spontaneous vagally mediated IBI organization in the study of sex differences.

One promising approach is the application of graph theory, a mathematical framework for nonlinear analysis. Widely used in neuroimaging to advance understanding of brain disorders [37, 39–41], it has also been applied to HRV, where methodological studies show it can effectively capture IBIs in short time segments, providing insight into moment-to-moment fluctuations in autonomic cardiac control [42, 43].

We have previously shown that a graph theory approach based on the similarity graph algorithm can assess IBI organization within short time windows defined to capture rapid vagal fluctuations [44]. In this context, nonlinearity refers to sensitivity to short-scale organization in IBIs that is not captured by averaged linear metrics. The similarity graph algorithm captures such features by quantifying interrelatedness among IBIs within brief, sliding time windows. The method [45] was superior to conventional vmHRV metrics in distinguishing adolescents with attention-deficit/hyperactivity disorder (ADHD) from healthy controls [44], with metrics derived from 2 to 5 s windows showing particular sensitivity—consistent with the time scale of vagal modulation.

**Fig. 1:**
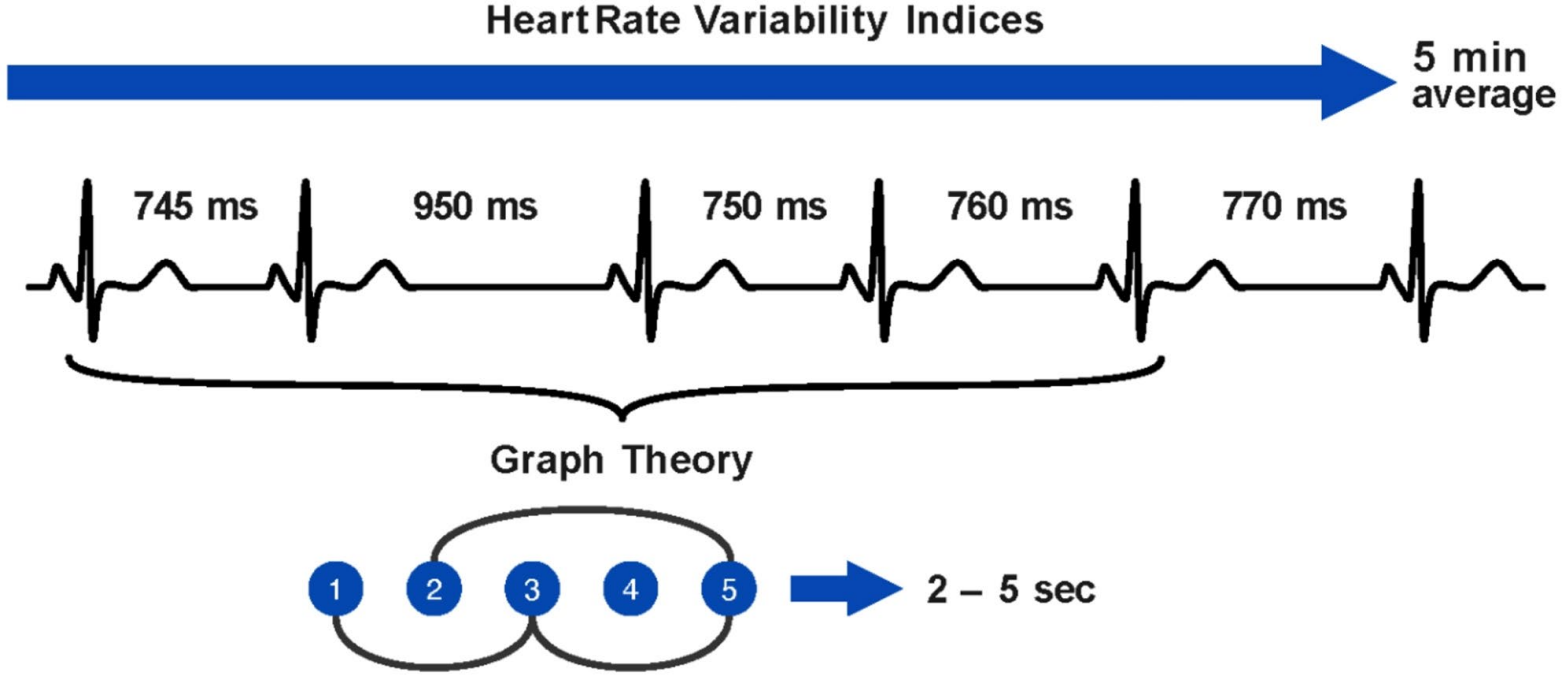
Illustration of nodes, edges, and time windows in the similarity graph approach. The upper panel shows a 5-minute IBI time series used to calculate HRV metrics. The lower panel illustrates a 2 + 2 neighbor subgraph based on five consecutive IBIs. Blue dots represent nodes, with the central node (“3”) as the index node. Edges (lines between nodes) are defined based on a similarity threshold of 1.5%. In this example, three edges are present and contribute to the total number of edges across all 2 + 2 neighbor subgraphs in the time series

The 2 + 2 neighbor subgraph corresponds to a time window of approximately 3 s, calculated from the combined duration of the five IBIs. For further details on the similarity graph approach, including node and edge construction and similarity thresholds, see the Methods section and [44].

The similarity graph algorithm has not yet been applied to young, healthy adults to examine how it may complement conventional vmHRV metrics in characterizing IBI organization and potential sex differences in vagal activity. In this approach, IBIs are represented as nodes in a network, with edges connecting similar values [46]. The number of neighboring IBIs included in each comparison is defined by the “neighbor” parameter (e.g., 2 + 2 neighbors correspond to a short, sliding time window of approximately 2–5 s, while 10 + 10 neighbors represent longer windows of 12–25 s), allowing the analysis of IBI organization across different temporal scales (see Fig. 1). The algorithm quantifies interrelatedness among IBIs, with a greater number of edges indicating higher similarity, which is typically associated with lower vmHRV [44]. It has also been successfully applied to capture short-term fluctuations in motor activity in bipolar disorder [45, 47] and reaction times in Parkinson’s disease [48].

In the current study, we applied the similarity graph algorithm to examine sex differences in IBI organization in young, healthy adults aged 18–30 years. We compared its sensitivity with conventional vmHRV metrics (lnRMSSD and lnHF-HRV). We hypothesized that the similarity graph metric edges 2 + 2 (i.e., the number of connections between adjacent inter-beat intervals in a 2 + 2 neighbor similarity graph) would be more sensitive in detecting sex differences, particularly by capturing spontaneous heart rate fluctuations within very short time windows (2–5 s), and would complement traditional HRV metrics when included in the same model. In line with previous literature [12], we further expected that males would show higher interrelatedness in IBIs (i.e., lower variability), reflected by a higher number of edges in the 2 + 2 time window and lower lnRMSSD and lnHF-HRV values.

## Methods

### Participants

A cross-sectional sample of 289 adults was pooled from three different projects [49–52], see Supplemental Fig. 1. The participants were university students from The Ohio State University (USA), the University of Bergen (Norway), and university colleges in Bergen (Norway). Participants were excluded due to missing information on age and sex (*n* = 2), being over 30 years (*n* = 14), and due to having extreme outlier scores on the graph theoretical metrics (*n* = 4; see Statistical analyses). The final sample consisted of 269 young, healthy adults aged 18 to 30 years old, with a mean age of 21.5 (SD = 3.0) and a comparable distribution of males and females (men = 141, 52.4%).

The sample was pooled from two projects (“Bergen 1” and “Bergen 2”) that were conducted at the University of Bergen, Norway. Bergen 1 contributed baseline data from a study on mindfulness training and psychophysiological flexibility [50, 51], approved by the Regional Ethics Committee in South-East Norway (2014/148). Bergen 2 contributed data from a study on emotion regulation and shame [52], approved by the Regional Ethics Committee in West Norway (33929). Additional data were taken from a third project conducted in the USA: the EDENS project (Ethnic Differences in the Experience of Noxious Stimuli), conducted at Ohio State University [49] and approved by the Institutional Review Board there (2017B0179). All participants were recruited through study advertisements and provided written informed consent prior to participation.

### Electrocardiogram (ECG) recordings and procedures

In the two Norwegian samples (Bergen 1 and Bergen 2), 5 min ECG recordings at rest were registered with the software Biopac Student Lab 4.0 (Biopac Systems Inc., Santa Barbara, CA) using hardware systems MP35 system (Bergen 1) and MP36 system (Bergen 2) with a lead-II configuration at a sampling rate of 1000 Hz. The registrations were done in a seated (Bergen 1) or in a supine (Bergen 2) position, with a period of 30 s in the same position prior to data acquisition in accordance with available recommendations [53]. The participants were asked to find a comfortable position and instructed to relax and breathe spontaneously. In the US EDENS study, 5 min ECG recordings at rest were registered with the software Biotrace+ (*Mind media B.V.*,* Roermond-Herten: Mind Media*,* BV*,* 2004*) using a 16-channel bioamplifier (*Nexus-16; Mind media B.V.; Roermon-Herten*,* The Netherlands*) with a sampling rate of 1000 Hz. The registration was done in a seated position, and the participants were instructed to relax and try not to fall asleep while breathing spontaneously.

### Heart rate variability metrics

Analyses of IBI organization and HRV metrics were performed using Kubios (versions 2.0/3.0) [54]. Data were first corrected for artifacts. In the Bergen 1 and 2 studies, RR interval series were visually inspected to identify artefactual beats (e.g., misplaced peaks, ectopic beats, and missed detections), which were manually corrected using cubic spline interpolation with default Kubios parameters [51]. In the US EDENS study, artifact correction was performed using Kubios’ automatic correction algorithm with a threshold of 0.45 s [49]. Detrending was applied using the smoothness prior method (λ = 500). RR intervals were linearly interpolated and resampled at 4 Hz prior to spectral analysis. Spectral estimation was performed using fast Fourier transform (FFT) with a Hanning window (256 points, 50% overlap) and autoregressive modeling (order 16). Frequency bands were defined as VLF (0–0.04 Hz), LF (0.04–0.15 Hz), and HF (0.15–0.40 Hz). All HRV metrics were calculated from normal-to-normal (NN) intervals, ensuring that analyses were restricted to normal sinus rhythm. Processed data were analyzed in Kubios to derive mean heart rate, ECG-derived respiratory rate (EDR), HF peak, and HRV metrics. The HRV metrics included in the current study (see Table 1) were natural log-transformed [54], including frequency-domain measures derived using fast Fourier transform (lnHF-HRV and lnLF-HRV) and the time-domain measure root mean square of successive differences (lnRMSSD) [20, 54]. Nonlinear metrics—sample entropy (SampEn), approximate entropy (ApEn), short-term (SD1) and long-term (SD2) variability (ms), and detrended fluctuation analysis, were computed in Kubios [54]. Entropy metrics (ApEn and SampEn) were calculated using an embedding dimension of m = 2 and a tolerance of *r* = 0.2 × SD of the RR interval series. DFA α1 was estimated over the short-term range (*n* = 4–12 beats) and α2 over the long-term range (*n* = 13–64 beats). These correspond to the default settings in Kubios HRV (versions 2.1 and 3), with identical algorithms and parameter settings across versions, ensuring comparability of nonlinear HRV indices across samples.

For HRV analyses, the full 5-minute recordings were used, as this is the standard and recommended duration [28, 55]. For the similarity graph theory algorithm (described below), 250 IBIs were analyzed to ensure an equal number of IBIs across participants for the calculation of edges 2 + 2 and edges 10 + 10. Previous work has shown that vmHRV metrics correlate highly (*r* = 0.82–0.97) when calculated from a fixed number of IBIs compared to full 5-minute recordings [44]. As the analysis windows are defined in beats rather than seconds, the effective time span varies between participants as a function of heart rate.

**Table 1:** Description of the similarity graph metrics and HRV metrics included in the current study

Measures	Indices	Description
Similarity graph theory	Edges 2 + 2	Number of edges connecting nodes in 2 + 2 neighbor graphs
	Edges 10 + 10	Number of edges connecting nodes in 10 + 10 neighbor graphs
Time-Domain HRV	lnRMSSD	Natural logarithm transformed values of square root of the mean squared differences between successive RR intervals (RMSSD) in ms
Frequency-Domain HRV	lnHF-HRV	Natural logarithm transformed values of absolute powers (ms^2^) of high frequency (HF) bands (0.15–0.40 Hz)
	lnLF-HRV	Natural logarithm transformed values of absolute powers (ms^2^) of low frequency (LF) bands (0.04–0.15 Hz)
	RESP	Respiration rate (derived from ECG and RR data)
Other Nonlinear HRV	SD1	Standard deviation perpendicular to the line of identity in the Poincaré plot
	SD2	Standard deviation along the line of identity in the Poincaré plot
	ApEn	Approximate entropy
	SampEn	Sample entropy
	DFA, α1	In detrended fluctuation analysis, short term fluctuation slope
Respiration	EDR	ECG derived respiration
	HF peak	Peak frequency within the high-frequency (HF) band

Higher scores (number of edges) for the similarity graph theory metrics indicate lower variability (and higher interrelatedness) in IBIs, whereas higher HRV scores reflect higher variability in IBIs

### The similarity graph theory algorithm

A heuristic algorithm was used to transform each IBI time series into a similarity graph G=(V, E) with nodes V and edges (this method is not chaos based) [45]. The program code is available at https://github.com/erlfas/SimilarityGraph and was implemented in Java, and has been used in previous studies applying the similarity graph theory algorithm [44, 45, 47, 48]. In this approach, every IBI in the time series is represented as a node in the graph. For a time series S, each node *u*_*i*_*in V*,* 1 ≤ I ≤ n*, corresponds to the element $x_i \in S$, and the node *u*_*i*_ is assigned a weight equal to *x*_*i*_.  

Similarity between two IBIs is determined using one of two criteria:

*max(x*_*i*_, *x*_*j*_*)/min(x*_*i*_, *x*_*j*_*) or max(x*_*i*_,*x*_*j*_*) − min(x*_*i*_, *x*_*j*_*)*. If the difference between two IBIs falls below a predefined threshold, an edge is drawn between the corresponding nodes. In this study, the similarity threshold was initially set to 1.5%, based on prior findings showing that this value best distinguished adolescents with ADHD from controls [44]. To assess robustness, the threshold was systematically varied from 0.5% to 4% (see Statistical Analyses and Results).

Each node serves as an index node that is analyzed in relation to surrounding nodes within a defined time window (see Fig. 1). The window size is specified by the number of neighboring nodes (*k*) on each side of the index node, giving a total of 2k neighbors. As the algorithm moves through the time series, the window slides and centers on each index node except the first *k* and last *k* IBIs. Different window sizes yield different subgraphs.

**Worked example.** For illustration, consider a short IBI sequence [800, 810, 790, 805, 795 ms]. Each IBI is represented as a node. Using a 2 + 2 window ($\:k=2$), the middle value (790 ms) serves as the index node, and its neighbors are 800, 810, 805, and 795 ms. With a similarity threshold of 1.5%, edges are drawn between the index node and neighboring nodes if their relative difference falls below this threshold. For example, the difference between 790 and 800 ms (≈ 1.27%) meets the criterion, and an edge is formed, whereas the difference between 790 and 810 ms (≈ 2.53%) exceeds the threshold and no edge is formed. This procedure is repeated for all valid index nodes as the window moves along the time series, and the number of edges is averaged across windows.

The primary outcome metrics were the number of edges detected within two window sizes: 2 + 2 neighbors and 10 + 10 neighbors. For each window, edges between the index node and its neighboring nodes were counted based on the similarity criterion and then averaged across the entire time series. Graphs with a higher number of edges (in both 2 + 2 and 10 + 10 windows) indicate greater similarity among IBIs, referred to here as higher interrelatedness.

The relationships between edges 2 + 2 and 10 + 10 and other similarity graph metrics (missing edges, bridges, and cliques; see [44]) are presented in Supplementary Table 4.

## Statistical analyses

All analyses were performed in SPSS version 31. Outliers in graph-theory and HRV metrics were identified using box plots, and extreme values exceeding ± 3 interquartile range (IQR) from the first or third quartile were removed due to potential artifacts. Independent samples t-test analyses were conducted to test for sex differences in age, body mass index (BMI), mean heart rate, respiration rate, or IBI. ANOVAs were conducted to examine whether there were significant differences in descriptive variables and graph-theory or HRV metrics among the three projects from which the data were pooled. Violin plots were used to visualize the distributions of the similarity graph metrics and HRV metrics. Skewness and kurtosis were examined, and variables were considered normally distributed when values were within ± 2. Descriptive statistics, including means, standard deviations (SDs), medians, and IQRs, were reported for the graph-theoretical and HRV metrics, together with the Common Language Effect Size (CLES). Pearson correlations were computed to assess associations between the graph metrics, HRV metrics, respiratory rate, mean heart rate, age, and BMI.

Multivariable logistic regression was conducted to examine whether lower IBI variability (i.e., higher interrelatedness) predicted sex (female = 0, male = 1). Model fit was evaluated using R² (Nagelkerke) and area under the curve (AUC). Effect sizes were expressed as odds ratios (ORs), with effects considered present when the OR and its 95% confidence interval exceeded 1. Two sets of models were estimated: (1) five separate models for each graph metric (edges 2 + 2, edges 10 + 10) and each HRV metric (lnRMSSD, lnHF-HRV, and lnLF-HRV), and (2) two combined models including graph metrics with predictive value together with lnRMSSD or lnHF-HRV. Due to multicollinearity, multiple graph metrics or multiple HRV metrics were not included in the same model. All models were adjusted for age and BMI. Follow-up models were additionally adjusted for project membership, and subsequently for respiratory rate and mean heart rate. Further, graph theory and HRV metrics were scaled to the IQR (adjusted for age and BMI), and logistic regression models were repeated with odds ratios representing a one-IQR increase (i.e., from the 1st to 3rd quartile). Because this analysis pooled all available observations from three suitable existing studies with accessible data, no a priori sample size calculation was performed. For the available sample size, we conducted a post hoc power assessment for the edges 2 + 2 metric using a simulation-based approach with 5,000 simulation runs (see Supplementary Methods for simulation details). Logistic regression was selected to enable direct comparison of the predictive value of graph-theory and HRV metrics within the same model, which would not be possible if these metrics were modeled as outcomes.

To assess the robustness of the 1.5% similarity threshold, the criterion was systematically varied from 0.5% to 4% for the graph theory metrics. Multivariable logistic regression analyses were used to evaluate whether alternative thresholds yielded stronger associations with sex. This range includes the commonly used sample entropy definition [56, 57], where the threshold is set to 20% of the IBI SD (0.20 × SD / mean), corresponding to approximately 1.5% in our previous study [44].

Supplementary analyses examined correlations between graph metrics and nonlinear HRV metrics (sample entropy, approximate entropy, SD1, SD2, and detrended fluctuation analysis), compared the predictive value of edges 2 + 2 with each nonlinear HRV index in multivariable models, and assessed correlations between edges 2 + 2 or edges 10 + 10 and additional graph metrics (missing edges, bridges, and cliques).

## Results

### Sample characteristics

Independent samples t-test analyses showed no statistically significant sex differences in age, BMI, mean heart rate, respiration rate, or IBI (see Supplemental Table 1). BMI ranged from 16 to 39 (M = 23.91, SD = 4.19). Mean heart rate (beats per minute) ranged from 51.82 to 106.61 (M = 73.17, SD = 10.18). Respiration rate was estimated using both EDR and HF peak frequency; EDR ranged from 0.01 to 0.39 (M = 0.21, SD = 0.06), and HF peak ranged from 0.15 to 0.38 (M = 0.22, SD = 0.06). IBI (ms) ranged from 567.70 to 1176.39 (M = 849.58, SD = 119.81).

Graph theory metrics and traditional HRV measures (lnRMSSD and lnHF-HRV) did not differ significantly across the three pooled projects (see Supplemental Table 2). lnLF-HRV was significantly higher in one project compared to the other two. The projects differed in age, sex distribution, BMI, EDR, HF peak, mean heart rate, and number of IBIs used for HRV calculations. However, after adjusting for these variables, no significant between-project differences remained for graph theory or vmHRV metrics, except for lnLF-HRV.

### Sex distribution of HRV and graph theory metrics

**Fig. 2:**
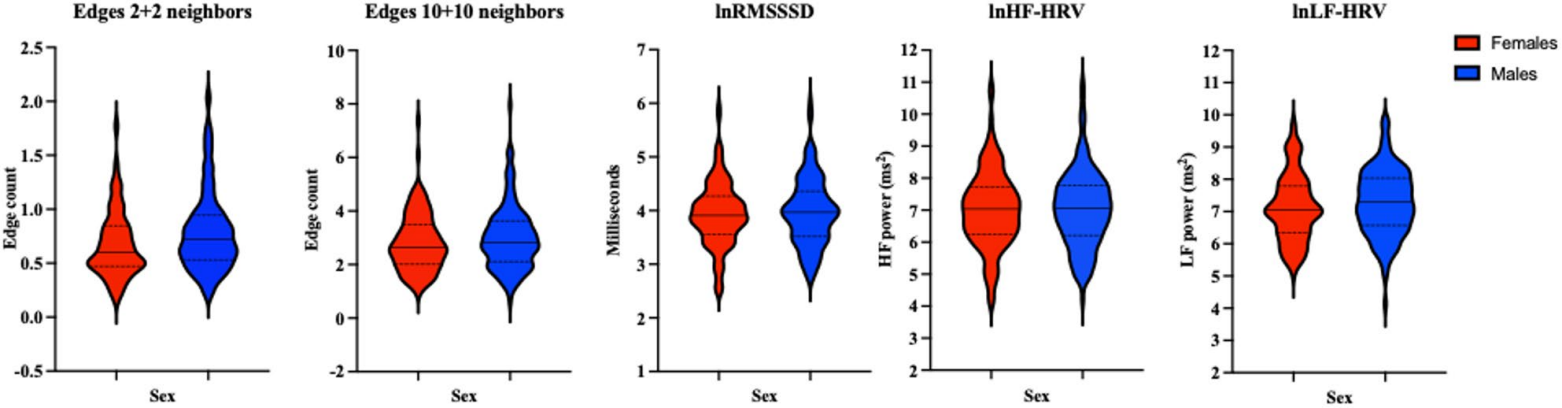
Violin plots showing the distributional patterns of the similarity graph metrics and HRV metrics

Figure 2 shows the distributions of the graph theory and HRV metrics. All variables fell within acceptable ranges for skewness and kurtosis. However, the edges 2 + 2 and edges 10 + 10 metrics were close to the upper limit for kurtosis (1.63 and 1.94, respectively).

Table 3 presents means, standard deviations, medians, and interquartile ranges for females and males. The CLES was highest for the edges 2 + 2 metric (CLES = 0.60), indicating a 60% probability that a randomly selected male has a higher value than a randomly selected female.

**Table 2:** Descriptive statistics for graph-theory and HRV metrics by sex

	Females				Males				CLES
	M	SD	Median	Q1-Q3	M	SD	Median	Q1-Q3	
Edges2 + 2	0.67	0.31	0.60	0.47–0.84	0.79	0.37	0.72	0.53–0.94	0.60
Edges10 + 10	2.82	1.07	2.64	2.01–3.50	2.99	1.18	2.81	2.11–3.63	0.54
lnRMSSD	3.94	0.60	3.91	3.56–4.27	3.98	0.63	3.97	3.52–4.36	0.52
lnHF-HRV	7.03	1.20	7.04	6.24–7.74	7.04	1.19	7.05	6.21–7.77	0.50
lnLF-HRV	7.12	1.03	7.04	6.34–7.79	7.28	1.02	7.29	6.57–8.04	0.54

IQR= Interquartile Range; CLES= Common Language Effect Size; Q= quartile; Q1-Q3 represent the first to third quartiles

### Bivariate correlations between HRV and graph theory metrics

Table 3 presents the Pearson correlation coefficients between the HRV metrics and the similarity graph metrics. The edges 2 + 2 and edges 10 + 10 metrics were strongly correlated with each other. Both metrics were also significantly associated with lower values of lnRMSSD, lnHF-HRV, and lnLF-HRV. None of the HRV or graph theory metrics showed significant correlations with age or BMI. All metrics were significantly correlated with respiratory rate as measured using the ERD, whereas only edges 10 + 10 and lnLF-HRV were correlated with respiratory rate when measured using the HF peak. Furthermore, all metrics were correlated with mean heart rate, with the exception of edges 10 + 10.

**Table 3:** Bivariate correlations between the HRV and graph theory metrics and these metrics’ correlations with age, BMI, ECG-derived respiration (EDR), and mean heart rate (M-HR)

		2.	3.	4.	5.	Age	BMI	EDR	HF peak	M-HR
1.	Edges 2 + 2	0.86**	− 0.45**	− 0.48**	− 0.26**	− 0.03	− 0.01	0.13*	-0.01	0.20**
2.	Edges 10 + 10		− 0.37**	− 0.40**	− 0.38**	− 0.01	− 0.01	0.19**	0.15*	0.07
3.	lnRMSSD			0.94**	0.67**	0.02	− 0.09	− 0.17**	0.04	− 0.51**
4.	lnHF-HRV				0.63**	0.01	− 0.09	− 0.14*	0.03	− 0.43**
5.	lnLF-HRV					0.03	− 0.09	− 0.32**	-0.15*	− 0.32**

*p = < 0.05; **p = < 0.001. BMI= body mass index; EDR = ECG derived respiration;

M-HR= mean heart rate

### The odds ratios of the HRV and graph theory metrics discriminating males from females

Multivariable logistic regression models were first estimated separately for each graph theory metric and index HRV (five models) due to multicollinearity, and thereafter two combined models compared graph theory metrics with vmHRV metrics (see Methods). Sex served as the outcome variable (female coded 0, male coded 1), and all models were adjusted for age and BMI. Overall, model fit was low for classifying sex based on graph-theory and HRV metrics, but models including the graph-theory metric edges 2 + 2 showed relatively better fit (see Table 4 for R^2^ values). The AUC value was highest for edges 2 + 2 (0.59) compared with edges 10 + 10 (0.54), lnRMSSD (0.51), lnHF-HRV (0.50), and lnLF-HRV (0.55).

**Fig. 3:**
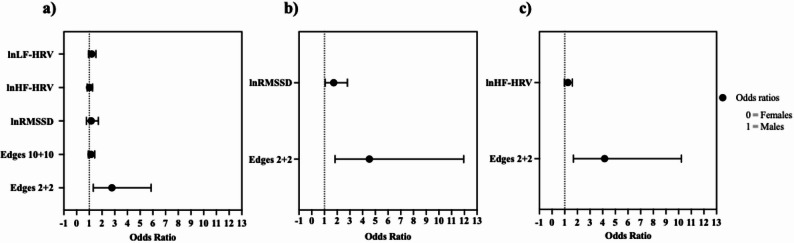
Odds ratios of the metrics scores of graph theory and HRV in discriminating males from females. In **a**), separate logistic regression models were conducted for each of the metrics as independent variables, whereas in **b**) and **c**), the 2 + 2 neighbor metrics were included in the same regression model as lnRMSSD or lnHF-HRV, respectively

Higher edges 2 + 2 values were associated with greater odds of being male, corresponding to roughly a threefold predicted increase for each unit of the metric (OR 2.78, 95% CI 1.32–5.86; see Table 4; Fig. 3a). When scaled to the interquartile range (IQR), keeping age and BMI constant, the edges 2 + 2 metric had an IQR of 0.39; a one-IQR increase (i.e., from the 1st to 3rd quartile) was associated with an odds ratio of 1.491 (95% CI 1.122–2.015; see Supplemental Table 3 for odds ratios per one-IQR increase). No other metrics produced odds ratios significantly different from 1. To address potential effects of combining data from three projects, project was added as a covariate in subsequent models. Project membership was not associated with sex classification (see Supplementary Table 4). Further, to assess whether mean heart rate and respiration acted as confounders, these variables were added as covariates and the logistic regression models were re-estimated. Their inclusion did not alter the predictive effects of the graph-theory or HRV metrics on sex (see Supplemental Table 5).

Two follow-up models tested whether edges 2 + 2 contributed additional explanatory value when entered together with lnRMSSD or lnHF-HRV. When combined with edges 2 + 2, lnRMSSD emerged as a significant predictor (OR 1.73, 95% CI 1.06–2.81; see Table 4; Fig. 3b), and the effect of edges 2 + 2 increased further (OR 4.53, 95% CI 1.83–11.20). In this combined model, a one-unit increase of edges 2 + 2 values was associated with a more than a fourfold increase in the predicted odds of being male compared with female. In contrast, lnHF-HRV remained nonpredictive, while edges 2 + 2 again demonstrated a significant association with sex (see Table 4; Fig. 3c). After adjustment for mean heart rate and respiratory rate, only edges 2 + 2 remained a significant predictor, whereas lnRMSSD was no longer associated with sex (see Supplemental Tables 5 and 6). Additional models indicated that this attenuation was primarily driven by adjustment for mean heart rate (see Supplemental Table 7, where respiration rate and mean heart rate were entered separately).

Post hoc power analysis indicated that an OR of 3.05 for the edges 2 + 2 metric would be required to achieve 80% statistical power for the given sample size in a logistic regression model including age and BMI as predictors of male sex. Assuming the observed OR of 2.78 reflects the true effect, the estimated statistical power was 74%.

To assess the robustness of the 1.5% similarity threshold for the edges 2 + 2 metric, multivariable logistic regression analyses adjusted for age and BMI were repeated using thresholds ranging from 0.5% to 4%. The commonly used criterion of 20% of the mean IBI standard deviation corresponded to a 2.8% threshold in the current study (0.20 × 119.81/849.58 ≈ 2.8%). and was included among the tested values. As shown in Supplemental Fig. 2, odds ratios increased with lower similarity thresholds, likely reflecting changes in the scaling of the IBI-derived metric and, consequently, the interpretation of a one-unit increase. Across thresholds, model performance remained similar, with R² values ranging from 0.048 to 0.053 and AUC values from 0.59 to 0.60. The 1.5% threshold yielded an AUC of 0.60.

**Table 4:** The odds ratios from the logistic regression models

			95% CI for OR				
			Odds ratios (OR)	Lower	Upper	*p*	*R* ^2^
Single-predictor models	Model 1	Age	0.972	0.895	1.056	0.508	0.051
		BMI	1.043	0.982	1.109	0.173	
		Edges 2 + 2	2.780*	1.318	5.864	0.007	
	Model 2	Age	0.970	0.894	1.053	0.473	0.021
		BMI	1.043	0.982	1.107	0.170	
		Edges 10 + 10	1.148	0.924	1.425	0.212	
	Model 3	Age	0.970	0.894	1.053	0.464	0.016
		BMI	1.044	0.983	1.108	0.159	
		lnRMSSD	1.153	0.775	1.713	0.483	
	Model 4	Age	0.970	0.894	1.053	0.470	0.014
		BMI	1.042	0.982	1.106	0.172	
		lnHF-HRV	1.021	0.834	1.251	0.837	
	Model 5	Age	0.969	0.893	1.052	0.456	0.022
		BMI	1.046	0.986	1.111	0.138	
		lnLF-HRV	1.195	0.941	1.516	0.144	
Combined-predictor models	Model 6	Age	0.975	0.896	1.060	0.548	0.075
		BMI	1.050	0.987	1.118	0.123	
		Edges 2 + 2	4.530*	1.833	11.195	0.001	
		lnRMSSD	1.725*^	1.059	2.810	0.028	
	Model 7	Age	0.976	0.898	1.061	0.572	0.066
		BMI	1.049	0.986	1.116	0.130	
		Edges 2 + 2	4.151	1.686	10.220	0.002	
		lnHF-HRV	1.248	0.974	1.600	0.080	

*ORs and 95% confidence intervals (CI) > 1 indicate increased odds. ^The prediction oflnRMSSD in model 6 did not exceed an OR and 95% CI of 1 when controlling for mean heart rate.Model fit was evaluated using Nagelkerke R². BMI= body mass index

### Supplementary analyses of other nonlinear HRV metrics and additional similarity graph theory metrics

Bivariate correlations showed that edges 2 + 2, lnRMSSD, and lnHF-HRV were significantly associated with all other nonlinear HRV metrics (ApEn, SD1, SD2, and DFA**α**1), except sample entropy (SamEn; see Supplementary Table 8). Edges 10 + 10 and lnLF-HRV correlated significantly with ApEn, SamEn, SD1, and SD2, but not with DFA**α**1.

Supplementary multivariable logistic regression analyses were then performed with each nonlinear HRV metric (SamEn, ApEn, SD1, SD2, and DFA**α**1) entered separately together with edges 2 + 2, adjusting for age and BMI. Edges 2 + 2 consistently was a statistically significant predictor of being male, whereas none of the nonlinear HRV metrics significantly predicted sex (see Supplementary Table 9). Model fit was evaluated using R² (Nagelkerke) and AUC (see Supplementary Table 9 for R^2^ values). The AUC value was highest for edges 2 + 2 (0.59) compared with SamEn (0.51), ApEn (0.49), SD1 (0.51), SD2 (0.50), and DFA**α**1 (0.56).

Bivariate correlations showed strong associations between edges 2 + 2 and 10 + 10 and the additional similarity graph metrics (number of missing edges, bridges, and cliques; see Supplementary Table 10). The HRV metrics lnRMSSD, lnHF-HRV, and lnLF-HRV also correlated significantly with these additional similarity graph metrics.

## Discussion

We found sex differences in very short-term heart rate fluctuations in young adults, specifically within 2–5-second time windows consistent with the timescale of parasympathetic (vagal) modulation of IBIs. Males showed greater interrelatedness in IBIs (i.e., lower HRV) than females, and only the similarity graph metric (edges 2 + 2) was associated with the odds of being male. The edges 2 + 2 metric appeared to capture rapid, spontaneous fluctuations more effectively than both the similarity graph metric based on longer time windows (≥12 s; edges 10 + 10) and standard vmHRV metrics. The similarity threshold was systematically varied, and 1.5% was retained for consistency with prior work and sensitivity to short-term IBI dynamics. Traditional vmHRV metrics (lnRMSSD, lnHF-HRV) did not differentiate between sexes on their own. However, when lnRMSSD was included in regression models together with edges 2 + 2, it discriminated between males and females only when mean heart rate was not controlled for; lnHF-HRV did not. There were no significant sex differences in mean heart rate, suggesting that lnRMSSD may still capture sex-related differences. Importantly, edges 2 + 2 remained predictive of sex differences after adjusting for respiratory rate and mean heart rate. Taken together, these findings suggest that sex differences in resting-state heart rate dynamics in young adults are primarily driven by rapid, spontaneous fluctuations in heart rate.

Although resting-state vmHRV metrics over several minutes are reliable trait markers of autonomic control [27], their relevance to dynamic, real-world conditions is sometimes questioned [58]. Nonlinear metrics provide complementary information by capturing rapid, spontaneous heart rate dynamics even at rest [37]. Rather than asking whether nonlinear methods add value, the key issue is which metrics best suit specific research or clinical aims.

Because cardiac regulation is highly complex, no single HRV index can fully represent autonomic function, underscoring the value of combining traditional and nonlinear metrics when examining sex differences in autonomic regulation [37]. However, commonly used nonlinear HRV metrics based on complexity or chaos theory do not specifically reflect vagal activity. In addition, some metrics, such as DFAα1, require relatively long recordings (> 5 min) for reliable estimation [59, 60], and metrics such as DFAα1 and entropy are typically computed as summary measures, limiting their ability to resolve moment-to-moment fluctuations within short (2–5 s) time windows. The similarity graph algorithm may therefore offer an alternative approach for capturing nonlinearity in vagally mediated IBIs.

The time-domain measure lnRMSSD complemented edges 2 + 2 in regression models, but this effect was attenuated after adjustment for mean heart rate, likely reflecting their shared variance. Given the well-established dependency between vmHRV and mean heart rate [61], this attenuation is expected. Although mean heart rate did not differ between sexes, lnRMSSD may still capture sex-related differences, but its independent contribution is reduced under such adjustment. In contrast, the association between edges 2 + 2 and sex remained unchanged after adjustment for both respiration rate and mean heart rate. This suggests that the similarity graph metric edges 2 + 2 captures more fine-grained, spontaneous IBI dynamics that are less influenced by physiological factors averaged over longer time scales.

The primary vmHRV metrics, lnRMSSD and lnHF-HRV, were not complemented to the same extent by edges 2 + 2. Only lnRMSSD improved prediction, and only in models without adjustment for mean heart rate, warranting cautious interpretation given their close relationship [61]. Although lnRMSSD and lnHF-HRV reflect overlapping short-term IBI dynamics, lnRMSSD may be more sensitive to rapid beat-to-beat fluctuations, aligning more closely with the dynamics captured by edges 2 + 2. This is consistent with evidence indicating that RMSSD can be estimated from shorter recordings, whereas HF-HRV typically requires longer segments due to its dependence on respiratory oscillations [62, 63]. Variability in breathing patterns may therefore have contributed to the weaker performance of lnHF-HRV. However, the validity and interpretation of ultra-short HRV measures remain debated [38, 64, 65], and further research is needed to clarify how similarity graph metrics relate to these measures.

Evidence suggests sex differences in adaptive self-regulation [13]. While psychophysiological markers may help clarify underlying mechanisms, these associations are indirect and should be interpreted with caution. vmHRV reflects aspects of autonomic regulation related to self-regulatory processes, but does not map directly onto behavioral or clinical outcomes. Differences in vagal activity may therefore contribute to, rather than fully explain, observed sex differences in self-regulation.

The present study focused on young adults because prior research suggests that sex differences in vagally mediated HRV are smaller in this age group. In contrast, such differences are more pronounced in both children and adolescents [11], and they re-emerge with increasing age in adulthood, particularly in lnRMSSD [12]. Interestingly, behavioral studies show a similar pattern: sex differences are reported more consistently in self-regulation before puberty, while findings in adult samples are more mixed [13]. Meta-analytic findings show that the HRV lifespan trajectory differs between sexes: adolescent males typically show higher HRV than females [11], whereas in adulthood females generally show higher HRV than males [12]. Thus, sex-specific changes in IBI regulation likely occur during the transition to young adulthood. Because adolescence typically ends around age 19 [66], with pubertal maturation occurring somewhat earlier [67], one would expect HRV sex differences to reverse in early adulthood—favoring females. Our findings support this pattern: in young adulthood, females showed lower interrelatedness in IBIs (i.e., higher HRV) compared with males.

Sex hormones may contribute to the lower vmHRV observed in males compared with females. In females, estradiol influences central autonomic pathways and may enhance parasympathetic modulation of cardiac activity [31, 68], whereas progesterone may be associated with increased sympathetic influences and cycle-related fluctuations [69], see also [70]. In males, testosterone has been linked to greater sympathetic influences, which may be associated with lower resting vmHRV. Importantly, vmHRV reflects the modulation of autonomic activity rather than the absolute level of cardiac vagal or sympathetic tone. Thus, higher vmHRV in females likely reflects greater modulation and adaptability of cardiac autonomic regulation rather than simply increased vagal tone. Sex hormones may also affect vmHRV indirectly via dopaminergic pathways, as estradiol modulates dopamine activity in brain regions involved in autonomic regulation, potentially supporting more dynamic and flexible cardiac control. These mechanisms may help explain the higher vmHRV typically observed in adult females, although further research is needed to clarify these interactions.

These mechanisms remain partly inferential, as the cross-sectional design precludes conclusions about longitudinal, sex-specific trajectories in heart rate fluctuations. In addition, information on menstrual cycle phase was not available, which may have introduced variability among female participants, given that HRV, particularly vagally mediated metrics such as lnRMSSD and lnHF-HRV, varies across the menstrual cycle and is recommended to be assessed during the follicular phase [68]. Although the relatively homogeneous sample may have reduced variability, future studies should consider controlling for or reporting menstrual cycle phase when examining sex differences in vmHRV. vmHRV is also influenced by dynamic factors such as activity and stress, which may affect its stability across days; however, repeatability could not be assessed in the present design. Furthermore, the reliability of the graph-theoretical metrics is not yet fully established, and the complex interplay between sympathetic and parasympathetic activity should be considered when interpreting the findings.

Although classification performance was modest, the aim of the study was to compare associations between variability metrics and sex rather than to develop predictive models. The post hoc power estimation indicated only slightly lower statistical power than the conventional threshold of 80%. Finally, pooling data from three projects limited the availability of common demographic variables, and the observed effects of sex were therefore not adjusted for several potential confounders, including educational level, medication use, smoking status, physical activity, dietary factors, hormonal contraceptive use, and environmental differences between study sites. However, the relatively homogeneous sample of healthy young adults may reduce the impact of these factors compared with more heterogeneous populations. In addition, although other HRV metrics such as pNN50 may be relevant in relation to graph-theoretical indices, lnRMSSD was used as the primary vagally mediated time-domain measure in accordance with established recommendations [18]. Respiration rate was estimated from EDR and HF peak, which provide indirect proxies of breathing and may be influenced by non-respiratory factors and signal variability.

Future research should employ longitudinal designs to examine sex differences in rapid, spontaneous heart rate fluctuations, including secondary analyses of existing datasets using the similarity graph algorithm. Incorporating respiration-guided HF-HRV analyses and measures of cardiorespiratory coupling may further clarify the relationship between respiratory dynamics and graph-theoretical metrics.

## Conclusions

This study examined the use of the similarity graph algorithm to capture rapid, spontaneous heart rate fluctuations. Our findings show that this method effectively detects sex differences in healthy young adults. We previously demonstrated that the algorithm also distinguishes adolescents with ADHD from those without more effectively than traditional HRV metrics [71]. By measuring heart rate fluctuations nonlinearly in very short time windows, the similarity graph algorithm aligns well with the timescale of spontaneous parasympathetic influences on heart rate.

## Supplementary Information

Below is the link to the electronic supplementary material.

Supplementary Material 1.

Supplementary Material 2.

Supplementary Material 3.

Supplementary Material 4.

## Data Availability

The dataset supporting the conclusions of this article is available at the Open Science Framwork (https://osf.io/pwv5u/overview?view_only=3ab687dfeb9a4a14894da71c49fcf952).
