# Supplementary material for: Capturing sex differences in spontaneous autonomic fluctuations of resting heart rate using a similarity graph theory approach
Source: Biol Sex Differ. 2026 Apr 25;17:119. doi: 10.1186/s13293-026-00904-x (PMC13262517; doi:10.1186/s13293-026-00904-x)
Supplement: Supplementary file 1 — Supplementary Material 1. [file 13293_2026_904_MOESM1_ESM.pdf]

Flow Chart for Sample Inclusion

1. Pooled Sample: N = 289

USA: EDENS
N = 147

Norway: Bergen 1
N = 62

Norway: Bergen 2
N = 80

2. Excluded Participants: n = 20

USA: EDENS

n = 2; no data on age and sex
n = 2: > 30 years old
n = 3: Extreme outliers on the graph theory indices

Norway: Bergen 1

n = 1: > 30 year old

Norway: Bergen 2

n = 11: > 30 years old
n = 1: Extreme outliers on the graph theory indices

3. Final Pooled Sample: N = 269

USA: EDENS
N = 140

Norway: Bergen 1
N = 61

Norway: Bergen 2
N = 68
