# Supplementary material for: Capturing sex differences in spontaneous autonomic fluctuations of resting heart rate using a similarity graph theory approach
Source: Biol Sex Differ. 2026 Apr 25;17:119. doi: 10.1186/s13293-026-00904-x (PMC13262517; doi:10.1186/s13293-026-00904-x)
Supplement: Supplementary file 2 — Supplementary Material 2. [file 13293_2026_904_MOESM2_ESM.docx]

**Supplementary Table 1.** Descriptive characteristics by sex.

**Supplementary Table 2.** Differences in descriptive variables, graph-theoretical metrics, and HRV metrics across the three pooled projects.

**Supplementary Table 3.** Odds ratios from logistic regression models based on interquartile range (IQR) scores.

**Supplementary Table 4.** Odds ratios from logistic regression models adjusted for project membership.

**Supplementary Table 5.** Odds ratios from logistic regression models adjusted for respiration rate and mean heart rate.

**Supplementary Table 6.** Odds ratios from logistic regression models adjusted for mean heart rate and two respiration rate metrics.

**Supplementary Table 7.** Odds ratios from logistic regression models adjusted for either mean heart rate or respiration rate (EDR or HF peak).

**Supplementary Table 8.** Bivariate correlations between graph theory metrics and linear and nonlinear HRV metrics.

**Supplementary Table 9.** The odds ratio from the logistic regression models of edges 2+2 and nonlinear HRV metrics.

**Supplementary Table 10.** Bivariate correlations between graph theory metrics, HRV metrics, and other graph-theoretical metrics not included in the current study.
