# Supplementary material for: Capturing sex differences in spontaneous autonomic fluctuations of resting heart rate using a similarity graph theory approach
Source: Biol Sex Differ. 2026 Apr 25;17:119. doi: 10.1186/s13293-026-00904-x (PMC13262517; doi:10.1186/s13293-026-00904-x)
Supplement: Supplementary file 3 — Supplementary Material 3. [file 13293_2026_904_MOESM3_ESM.pdf]

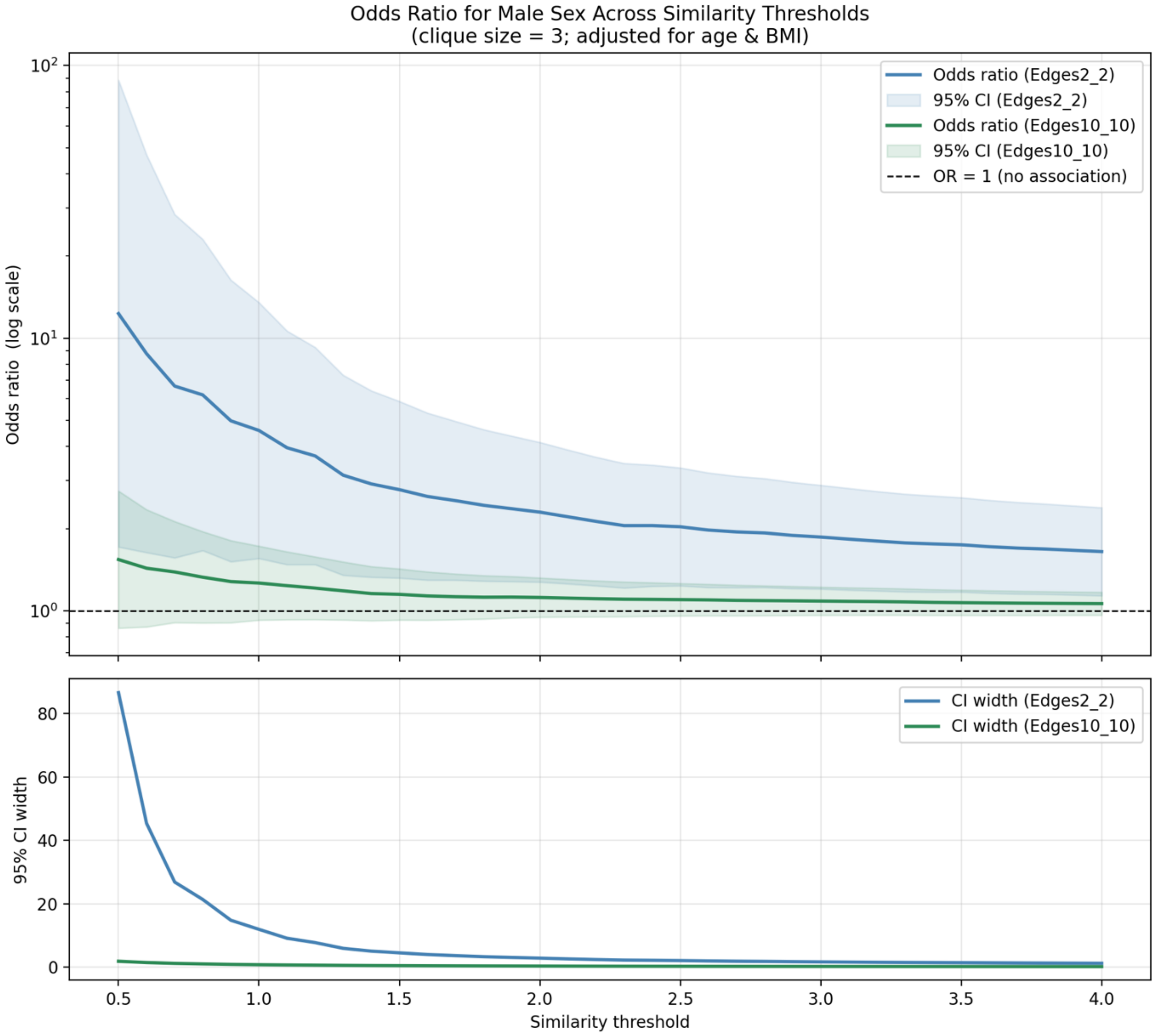

Supplemental Figure 2. Upper panel: Odds ratios across different similarity thresholds. Lower panel: corresponding 95% confidence intervals. Blue lines represent the Edges 2+2 metric, and green lines represent the Edges 10+10 metric. Odds ratios for Edges 2+2 increased with lower similarity thresholds, likely reflecting changes in scaling and the interpretation of a one-unit increase in the IBI-derived metric. Model performance remained similar across thresholds ($R^2 = 0.048–0.053$; AUC = 0.59–0.60), with the 1.5% threshold yielding an AUC of 0.60.
