# Supplementary material for: Capturing sex differences in spontaneous autonomic fluctuations of resting heart rate using a similarity graph theory approach
Source: Biol Sex Differ. 2026 Apr 25;17:119. doi: 10.1186/s13293-026-00904-x (PMC13262517; doi:10.1186/s13293-026-00904-x)
Supplement: Supplementary file 4 — Supplementary Material 4. [file 13293_2026_904_MOESM4_ESM.docx]

**Supplementary Methods for Post Hoc Power Estimation**

For the given sample size of 269, we performed a post-hoc assessment of statistical power for the edges 2+2 metric with a simulation approach based on 5000 simulation runs. For each simulation run, we first generated synthetic data for age, BMI, and the edges 2+2 metric by randomly drawing from parametric distributions that were similar to the respective observed distributions: Exponential for age, lognormal for BMI and edges 2+2. While the true odds ratios of age and BMI were fixed to their respective empirically observed odds ratios, the odds ratio for the edges 2+2 metric was systematically varied. Predicted probabilities of being male were derived from the inverse logit function applied to the predicted logits (the linear predictor in the logistic regression model), and events of being male were simulated by drawing from a binomial distribution. P-values were derived from the likelihood ratio test of the logistic regression with age, BMI and the edges 2+2 metric against the model with only age and BMI. Statistical power was estimated as the proportion of statistically significant simulation runs.

Sample observed proportion male: 0.52416

Sample observed Nagelkerke R^2^ Edges 2+2: 0.05085

Number of simulation runs: 5000

Simulation: mean proportion male: 0.52233

Simulation: mean Nagelkerke R^2^ Edges 2+2: 0.05555

Simulation: N=264, power for observed Odds Ratio = 2.783: 0.732

Simulation: N=264, power for Odds Ratio = 3.05: 0.8064
